# Homologous or heterologous booster of inactivated vaccine reduces SARS-CoV-2 Omicron variant escape from neutralizing antibodies

**DOI:** 10.1080/22221751.2022.2030200

**Published:** 2022-02-04

**Authors:** Xun Wang, Xiaoyu Zhao, Jieyu Song, Jing Wu, Yuqi Zhu, Minghui Li, Yuchen Cui, Yanjia Chen, Lulu Yang, Jun Liu, Huanzhang Zhu, Shibo Jiang, Pengfei Wang

**Affiliations:** a State Key Laboratory of Genetic Engineering, Shanghai Institute of Infectious Disease and Biosecurity, School of Life Sciences, Fudan University, Shanghai, People’s Republic of China; b Department of Infectious Diseases, Huashan Hospital affiliated with Fudan University, Shanghai, People’s Republic of China; c State Key Laboratory of Genetic Engineering and Engineering Research Center of Gene Technology, Ministry of Education, Institute of Genetics, School of Life Sciences, Fudan University, Shanghai, People’s Republic of China; d Fubio (Suzhou) Biomedical Technology Co., Ltd., Suzhou, People’s Republic of China; e Key Laboratory of Medical Molecular Virology (MOE/NHC/CAMS), School of Basic Medical Sciences, Shanghai Institute of Infectious Disease and Biosecurity, Fudan University, Shanghai, People’s Republic of China

**Keywords:** SARS-CoV-2, Omicron, convalescent, inactivated vaccine, monoclonal antibodies

## Abstract

The massive and rapid transmission of SARS-CoV-2 has led to the emergence of several viral variants of concern (VOCs), with the most recent one, B.1.1.529 (Omicron), which accumulated a large number of spike mutations, raising the specter that this newly identified variant may escape from the currently available vaccines and therapeutic antibodies. Using VSV-based pseudovirus, we found that Omicron variant is markedly resistant to neutralization of sera from convalescents or individuals vaccinated by two doses of inactivated whole-virion vaccines (BBIBP-CorV). However, a homologous inactivated vaccine booster or a heterologous booster with protein subunit vaccine (ZF2001) significantly increased neutralization titers to both WT and Omicron variant. Moreover, at day 14 post the third dose, neutralizing antibody titer reduction for Omicron was less than that for convalescents or individuals who had only two doses of the vaccine, indicating that a homologous or heterologous booster can reduce the Omicron escape from neutralizing. In addition, we tested a panel of 17 SARS-CoV-2 monoclonal antibodies (mAbs). Omicron resists seven of eight authorized/approved mAbs, as well as most of the other mAbs targeting distinct epitopes on RBD and NTD. Taken together, our results suggest the urgency to push forward the booster vaccination to combat the emerging SARS-CoV-2 variants.

## Introduction

Coronavirus disease 2019 (COVID-19), caused by severe acute respiratory syndrome coronavirus 2 (SARS-CoV-2), continues to disrupt worldwide social and economic equity, and global public health. As of December 2021, more than 278 million confirmed cases of COVID-19, including 5.4 million deaths, have been reported across the world (https://www.worldometers.info/coronavirus). The massive and rapid transmission of SARS-CoV-2 has led to the emergence of several viral variants, some of which have raised high concern due to their impact on transmissibility, mortality and putative capacity to escape from immune responses generated after infection or vaccination [1]. Distributed throughout the world, the previous four well-characterized SARS-CoV-2 variants of concern (VOCs), including Alpha (B.1.1.7), Beta (B.1.351), Gamma (P.1), and Delta (B.1.617.2), are responsible for a second and third wave of the pandemic [1,2]. Of note, the Beta variant displayed the greatest magnitude of immune evasion from both serum neutralizing antibodies and therapeutic monoclonal antibodies, whereas Delta exhibited even greater propensity to spread coupled with a moderate level of antibody resistance [3–6].

Recently, a new variant of SARS-CoV-2, Omicron (B.1.1.529), was first reported to the World Health Organization (WHO) by South Africa on November 24, 2021. It has been rapidly spreading and was immediately designated as a VOC by WHO within two days [7,8]. Strikingly, analysis of the genomic sequences of the Omicron variant revealed that its spike harbours a high number of mutations, including 15 mutations in the receptor-binding domain (RBD), raising the specter that this newly identified variant may escape from the currently available vaccines and therapeutic antibodies. Moreover, the new variant shares several mutations with the previous VOC Alpha, Beta, Gamma and Delta variants, which further raised global concerns about its transmissibility, pathogenicity, and immune evasion. Here in this study, we constructed the Omicron pseudovirus (PsV) and tested its neutralization sensitivity against convalescent and vaccinee sera as well as a panel of monoclonal antibodies (mAbs).

## Results

**Figure 1.**
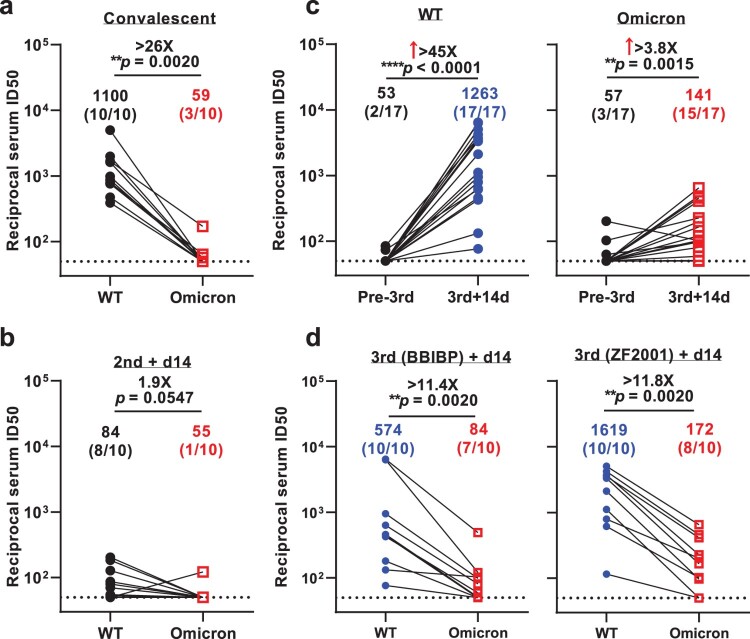
Neutralization of pseudotyped WT (D614G) and Omicron (B.1.1.529) viruses by convalescent sera (**a**), sera collected at day 14 post 2-dose BBIBP-CorV (**b**), sera collected before vs. at day 14 post the booster dose (**c**), and sera collected at day 14 post-BBIBP-CorV or -ZF001 booster dose (**d**). For all panels, values above the symbols denote geometric mean titer and the numbers in parentheses denote the proportion of positive sera with ID_50_ above the LOQ (dotted lines, >1:50). *P* values were determined by using a Wilcoxon matched-pairs signed-rank test (two-tailed).

A key question in the Omicron investigation thus far is its putative ability to escape immune surveillance. Therefore, we took steps to measure and clarify the extent of such immune evasion by this VOC after infection or vaccination. To accomplish this, we first evaluated the neutralizing activity of serum collected from 10 convalescent patients infected with the Delta variant of SARS-CoV-2 (Supplementary Table 1). Using VSV-based PsVs, we observed robust titers against WT virus in all 10 sera, with the geometric mean neutralizing titers (GMTs) of about 1100. However, the Omicron variant was markedly resistant to neutralization by convalescent plasma. Only 3 out of the 10 sera showed ID50 titers above the lower limit of quantification (LOQ) and a significant drop (>26-fold) compared WT was observed (Figure 1(a) and Supplementary Figure 1(a)). We then assessed the neutralizing activity of sera from individuals vaccinated by two doses of inactivated whole-virion vaccines (BBIBP-CorV) (Supplementary Table 1). We found that the GMT against WT was 84 with 80% vaccinees showing positive neutralization activity, while only 10% vaccinees showed successful neutralization against Omicron (Figure 1(b) and Supplementary Figure 1(b)). This first impression of Omicron’s immune evasion capability was indeed striking.

Then we turned our attention to the efficacy of booster shots now routinely administered in many countries and reported to induce higher immune response [9]. This time, to measure and clarify the efficacy of vaccine boosters against the Omicron variant, we collected samples from healthy adults who had a third boosting vaccination shot with either an inactivated whole-virion vaccine (BBIBP-CorV, homologous booster group) or a protein subunit vaccine (ZF2001, heterologous booster group) administered at an interval of 4–8 months following previous priming vaccination by two doses of BBIBP-CorV vaccine (Supplementary Table 1). Here, we found that booster vaccine significantly increased the titer of neutralizing antibodies against both the WT and Omicron viruses. For the WT virus, the booster improved the neutralization titer more than 45-fold (GMT = 1263), and for Omicron, the titer increased about 4-fold (GMT = 141, Figure 1(c) and Supplementary Figure 1(c–d)). At day 14 post the booster dose, whether homologous or heterologous, the neutralization titer for Omicron was significantly reduced for both the homologous and heterologous booster, about 11-fold in comparison to WT. However, the reduction fold was less than that for convalescents and the proportion of sera retain active was higher than that of individuals who had only two doses of inactivated vaccine (70–80% vs. 10%) (Figure 1(d) and Supplementary Figure 1(d)). After we carried out a comparison between the homologous and heterologous booster groups, we found that there was no significant difference regarding their neutralization titers against WT strain and Omicron variant, or the fold of reduction of neutralization against WT strain and Omicron variant (Supplementary Figure 2). These results indicate that either homologous or heterologous booster can reduce the Omicron escape from neutralizing antibodies.

**Figure 2.**
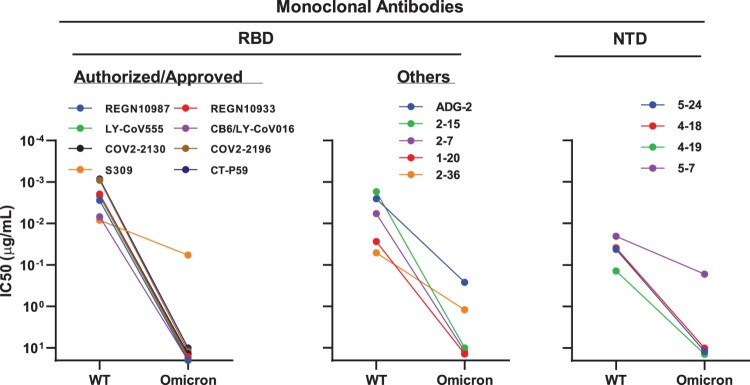
Neutralization of pseudotyped WT (D614G) and Omicron (B.1.1.529) viruses by mAbs targeting different epitopes.

To understand which types of antibodies in serum lose their activity against Omicron, we further evaluated the neutralization profile of a panel of mAbs targeting SARS-CoV-2 spike (Supplementary Figure 3). These mAbs included most that have been authorized or approved for clinical use: REGN10987 (imdevimab) [10], REGN10933 (casirivimab) [10], LY-CoV555 (bamlanivimab) [11], CB6/LY-CoV016 (etesevimab) [12], S309 (sotrovimab) [13], COV2-2130 (cilgavimab) [14], COV2-2196 (tixagevimab) [14], and CT-P59 (regdanvimab) [15], all of which are directed to RBD. Here, we found that seven out of the eight mAbs tested completely lost their neutralizing activity against the Omicron variant. The only approved antibody that retained its neutralizing activity was S309, but still had a 7-fold reduction compared to WT (Figure 2, left panel). We further tested some other RBD-directed mAbs of interest, including ADG-2 [16], which is now under development by Adagio Therapeutics, and four more antibodies from our own collection, including 1–20 [17], 2–15 [17], 2–7 [17], and 2–36 [17,18], belong to class 1–4, respectively. The inability of these RBD mAbs to match the risk posed by Omicron was again apparent since all five mAbs tested lost their neutralizing activity, either completely (1–20, 2–15, and 2–7), or partially (ADG-2 and 2-36) (Figure 2, middle panel). We then assessed the neutralizing activity of four N-terminal domain (NTD)-directed mAbs against Omicron and WT viruses, including three targeting the antigenic supersite (5–24, 4–18, and 4–19) [17,19] and one targeting a distinct site on the NTD (5–7) [17,20]. As shown in the right panel of Figure 2, the activity against Omicron of the supersite-directed mAbs was totally abolished, but only 5–7 retained its activity partially.

## Discussion

The SARS-CoV-2 Omicron variant struck the world soon after its identification. The retrospective analysis of epidemiological surveillance data in South Africa showed that Omicron is associated with a higher rate of reinfection [21]. Besides, the protection against infection of Omicron from symptomatic disease at 25 weeks after 2-dose vaccination was suggested to be less than 10% in the UK [22]. Similar to these preliminary real-world data, here we reported that a markedly reduced neutralizing activity against Omicron variant in the convalescent and two-dose BBIBP-CorV vaccination group, which is also confirmed by others [23–26]. However, the good news is that after two doses of inactivated vaccines as the “priming” shot, a third homologous inactivated vaccine booster or a heterologous protein subunit vaccine booster could elevate neutralization titer against Omicron. Our results are quite comparable to studies on the same inactivated vaccine [23] or other vaccines [27], and again supported by the real-world data showing a third dose booster vaccination provides increased protection against Omicron [28].

We further investigated the immune evasion capacity of Omicron with mAbs targeting different regions of viral spike protein. Comparable to others [25,29–31], our data suggest the substantial loss of neutralizing activity against Omicron by many mAbs, however, there might be still some conserved sites in RBD (S309 site [13]) or NTD (5–7 site [20]) that could be targeted by antibodies. A vaccine booster, either homologous or heterologous, is expected to elicit such neutralizing antibodies that help reduce the Omicron variant escape and improve the protection. Therefore, it is advisable to push forward booster shots where conditions permit.

## Methods

### Serum samples

Convalescent plasma samples (*n* = 10) were obtained from patients after 3–4 months of SARS-CoV-2 breakthrough infection caused by Delta variant in July 2021. Among them, nine participants were immunized with two-dose inactivated vaccines (CoronaVac) pre-infection. Sera from individuals who received two or three doses of BBIBP-CorV or ZF2001 vaccine were collected at Huashan Hospital, Fudan University 14 days after the final dose. All collections were conducted according to the guidelines of the Declaration of Helsinki and approved by the Institutional Review Board of the Ethics Committee of Huashan Hospital (2021-041 and 2021-749). All the participants provided written informed consents.

### Monoclonal antibodies

Monoclonal antibodies tested in this study were constructed and produced at Fudan University.

### Construction and production of variant pseudoviruses

Plasmids encoding the WT (D614G) SARS-CoV-2 spike and Omicron variant (B.1.1.529) spike were synthesized. Expi293 cells were grown to 3×10^6^/mL before transfection with the spike gene using Polyethylenimine (Polyscience). Cells were cultured overnight at 37°C with 8% CO_2_ and VSV-G pseudo-typed ΔG-luciferase (G*ΔG-luciferase, Kerafast) was used to infect the cells at a multiplicity of infection of five for 4 h before washing the cells with 1×DPBS three times. The next day, the transfection supernatant was collected and clarified by centrifugation at 300 g for 10 min. Each viral stock was then incubated with 20% I1 hybridoma (anti-VSV-G; ATCC, CRL-2700) supernatant for 1 h at 37 °C to neutralize the contaminating VSV-G pseudotyped ΔG-luciferase virus before measuring titers and making aliquots to be stored at −80 °C.

### Pseudovirus neutralization assays

Neutralization assays were performed by incubating pseudoviruses with serial dilutions of monoclonal antibodies or sera, and scored by the reduction in luciferase gene expression. In brief, Vero E6 cells were seeded in a 96-well plate at a concentration of 2×10^4^ cells per well. Pseudoviruses were incubated the next day with serial dilutions of the test samples in triplicate for 30 min at 37 °C. The mixture was added to cultured cells and incubated for an additional 24 h. The luminescence was measured by Luciferase Assay System (Beyotime). IC_50_ was defined as the dilution at which the relative light units were reduced by 50% compared with the virus control wells (virus + cells) after subtraction of the background in the control groups with cells only. The IC_50_ values were calculated using nonlinear regression in GraphPad Prism.

## Supplementary Material

Supplemental Material

## Data Availability

Materials used in this study will be made available but may require execution of a materials transfer agreement. All the data are provided in the paper or the Supplementary Information.
