# Supplementary material for: Homologous or heterologous booster of inactivated vaccine reduces SARS-CoV-2 Omicron variant escape from neutralizing antibodies
Source: Emerg Microbes Infect. 2022 Feb 4;11(1):477–81. doi: 10.1080/22221751.2022.2030200 (PMC8820826; doi:10.1080/22221751.2022.2030200)
Supplement: Supplemental Material [file TEMI_A_2030200_SM5763.docx]

**Supplementary Material for**

**Homologous or Heterologous Booster of Inactivated Vaccine Reduces SARS-CoV-2 Omicron Variant Escape from Neutralizing Antibodies**

Xun Wang^1,#^, Xiaoyu Zhao^1,#^, Jieyu Song^2,#^, Jing Wu^2,#^, Yuqi Zhu^3,#^, Minghui Li^1^, Yuchen Cui^1^, Yanjia Chen^1^, Lulu Yang^1^, Jun Liu^3,4^, Huanzhang Zhu^3^, Shibo Jiang^5^, Pengfei Wang^1,3,*^

^1^Shanghai Institute of Infectious Disease and Biosecurity, School of Life Sciences, Fudan University, Shanghai 200438, China

^2^Department of Infectious diseases, Huashan Hospital affiliated to Fudan University, Shanghai 200040, China

^3^State Key Laboratory of Genetic Engineering and Engineering Research Center of Gene Technology, Ministry of Education, Institute of Genetics, School of Life Sciences, Fudan University, Shanghai 200438, China

^4^Fubio (Suzhou) Biomedical Technology Co., Ltd.

^5^Key Laboratory of Medical Molecular Virology (MOE/NHC/CAMS), School of Basic Medical Sciences, Shanghai Institute of Infectious Disease and Biosecurity, Fudan University, Shanghai 200032, China

^#^These authors contributed equally

^*^Correspondence and requests for materials should be addressed to Pengfei Wang ([pengfei_wang@fudan.edu.cn](mailto:pengfei_wang@fudan.edu.cn)).

Supplementary Information and Figures

Supplementary Figure 1. Neutralization curves for convalescent sera (a), sera collected at day 14 post the second dose of BBIBP-CorV (b), sera collected before the booster dose (c), and sera collected at day 14 post the BBIBP-CorV or ZF001 booster dose (d).

Supplementary Figure 2. Comparison between the BBIBP-CorV (homologous) and ZF2001 (heterologous) booster groups on their neutralization titers for WT (a) or Omicron (b) viruses, or their reduction levels comparing Omicron from WT(c).

Supplementary Figure 3. Neutralization curves for mAbs.

**Supplementary Table 1.** Bassline characteristics of enrolled participants, including convalescent patients, BBIBP-CorV two doses group, BBIBP-CorV homologous booster group and BBIBP-CorV/ ZF2001 heterologous booster group.

Supplementary Figures

**Supplementary Figure 1.** Neutralization curves for convalescent sera (**a**), sera collected at day 14 post- second dose of BBIBP-CorV (**b**), sera collected before booster dose (**c**), and sera collected at day 14 post the BBIBP-CorV or ZF001 booster dose (**d**).

**Supplementary Figure 2.** Comparison between the BBIBP-CorV (homologous) and ZF2001 (heterologous) booster groups on their neutralization titers for WT (**a**) or Omicron (**b**) viruses, or their reduction levels comparing Omicron from WT(**c**).

**Supplementary Figure 3. Neutralization curves for mAbs.**

**Supplementary Table 1.** Bassline characteristics of enrolled participants, including convalescent patients, BBIBP-CorV two doses group, BBIBP-CorV homologous booster group and BBIBP-CorV/ZF2001 heterologous booster group.

|  | **Convalescent patients**  (n=10) | **BBIBP-CorV two doses group**  (n=10) | **BBIBP-CorV homologous booster group** (n=10) | **BBIBP-CorV/ ZF2001 heterologous booster group** (n=10) | **P value** |
| --- | --- | --- | --- | --- | --- |
| **Age (years), median(range)** | 46 (34-54) | 33.5 (22-47) | 26(19-31) | 29.5(23-56) | <0.0001 |
| **Male, n (%)** | 3 (30.00%) | 5 (50.00%) | 6 (60.00%) | 45 (50.00%) | 0.684 |
| **BMI (kg/m^2^), mean (SD)** | 24.45(5.64) | 22.81 (3.27) | 21.61 (3.10) | 21.66 (3.36) | 0.352 |
| **Comorbidities (%)** |  |  |  |  |  |
| Any, n (%) | 4 (40.00%) | 0 (0.00%) | 0 (0.00%) | 0 (0.00%) | 0.009 |
| Cardiovascular diseases, n (%) | 0 (0.00%) | 0 (0.00%) | 0 (0.00%) | 0 (0.00%) | - |
| Hypertension, n (%) | 3 (30.00%) | 0 (0.00%) | 0 (0.00%) | 0 (0.00%) | 0.049 |
| Diabetes, n (%) | 1 (10.00%) | 0 (0.00%) | 0 (0.00%) | 0 (0.00%) | 1.000 |
